# Supplementary material for: Mirages in continuous directed enzyme evolution: a cautionary case study with plantized bacterial THI4 enzymes
Source: Plant Biotechnol J. 2025 Jan 3;23(4):1070–2. doi: 10.1111/pbi.14563 (PMC11933848; doi:10.1111/pbi.14563)
Supplement: Supplementary file 2 — Figure S1. Confirmation of the effectiveness of the expression‐reduction regimes using yeast THI4. [file PBI-23-1070-s002.docx]

Empty vector Yeast THI4

7.0

6.0

5.0

4.0

3.0

2.0

1.0

0.0

72A 24A

611 611

0A

611

72A 72A 24A

633

611

611

0A 72A

611 633

OD600 at day 5

**Figure S1** Confirmation of the effectiveness of the expression-reduction regimes using yeast THI4. The native yeast THI4 gene was cloned into p1 with a poly(A) tail of 72A to 24A or 0A and replicated by TP-DNAP1_611 (611), or with a 72A tail and replicated by TP-DNAP1_633 (633). Each combin- ation was accompanied by an empty vector control. The growth medium was thiamin- and HET-free. Data are means and SE for triplicate cultures at 5 days. Note that the expression-reduction regimes all suppressed growth as expected, whereas the 72A / 611 benchmark combination grew normally.
