# Supplementary material for: Mirages in continuous directed enzyme evolution: a cautionary case study with plantized bacterial THI4 enzymes
Source: Plant Biotechnol J. 2025 Jan 3;23(4):1070–2. doi: 10.1111/pbi.14563 (PMC11933848; doi:10.1111/pbi.14563)
Supplement: Supplementary file 3 — Table S1. Conservation of MhTHI4 V124A mutated residues among representative prokaryotic THI4s. [file PBI-23-1070-s001.docx]

**Table S1.** Conservation of MhTHI4 V124A mutated residues among representative prokaryotic THI4s

Diverse THI4 sequences from 199 genomes (Joshi *et al*., 2021) were aligned and compared to the nonsynonymous mutations in MhTHI4. Natural replacements corresponding to mutations are in red.

Mutation Natural replacements

F14L **L** Y Q A H M S I T K V N

I27T V L F

L35S I M F V

Y40H **H** K A E F D R V N T

T117A **A** R K V M H L Q S N E I

Y122C T S F M V I L W A

A124V **V** I M F L

V127A **A** I L

I198T V M A C P S I

E202G D

K244E **E** Q R L A
